# Gazing Beyond the Horizon: A Systematic Review Unveiling the Theranostic Potential of Quantum Dots in Alzheimer's Disease

**DOI:** 10.7759/cureus.58677

**Published:** 2024-04-21

**Authors:** Tanya Sinha, Syed Faqeer Hussain Bokhari, Muhammad Usman Khan, Muhammad Sarim Shaheer, Maaz Amir, Beenish Fatima Zia, Danyal Bakht, Muhammad Arsham Javed, Mohammed Khaleel I.KH. Almadhoun, Mohammad Burhanuddin, Sai Teja Puli

**Affiliations:** 1 Medical Education, Tribhuvan University, Kathmandu, NPL; 2 Medicine and Surgery, King Edward Medical University, Lahore, PAK; 3 Internal Medicine, Faisalabad Medical University, Faisalabad, PAK; 4 Biochemistry, ABWA Medical College, Faisalabad, PAK; 5 Medicine, Fatima Memorial Hospital College of Medicine and Dentistry, Lahore, PAK; 6 Medicine and Surgery, Mutah University, Karak, JOR; 7 Medicine, Bhaskar Medical College, Hyderabad, IND; 8 Internal Medicine, Bhaskar Medical College, Hyderabad, IND

**Keywords:** nanotechnology, drug delivery, biomarkers, nanotheranostics, neuroinflammation, tau protein, amyloid-beta, theranostics, quantum dots, alzheimer's disease

## Abstract

Alzheimer's disease (AD), a neurodegenerative disorder characterized by cognitive decline, poses a significant healthcare challenge worldwide. The accumulation of amyloid-beta (Aβ) plaques and hyperphosphorylated tau protein drives neuronal degeneration and neuroinflammation, perpetuating disease progression. Despite advancements in understanding the cellular and molecular mechanisms, treatment hurdles persist, emphasizing the need for innovative intervention strategies. Quantum dots (QDs) emerge as promising nanotechnological tools with unique photo-physical properties, offering advantages over conventional imaging modalities. This systematic review endeavors to elucidate the theranostic potential of QDs in AD by synthesizing preclinical and clinical evidence. A comprehensive search across electronic databases yielded 20 eligible studies investigating the diagnostic, therapeutic, or combined theranostic applications of various QDs in AD. The findings unveil the diverse roles of QDs, including inhibiting Aβ and tau aggregation, modulating amyloidogenesis pathways, restoring membrane fluidity, and enabling simultaneous detection of AD biomarkers. The review highlights the potential of QDs in targeting multiple pathological hallmarks, delivering therapeutic payloads across the blood-brain barrier, and facilitating real-time imaging and high-throughput screening. While promising, challenges such as biocompatibility, surface modifications, and clinical translation warrant further investigation. This systematic review provides a comprehensive synthesis of the theranostic potential of QDs in AD, paving the way for translational research and clinical implementation.

## Introduction and background

Alzheimer's disease (AD) holds prominence as the primary cause of dementia in the elderly population, affecting a substantial segment of individuals aged 65 years and older worldwide [1]. This neurodegenerative ailment unfolds progressively, intricately intertwined with cognitive and behavioral dysfunctions. Its onset, often subtle, marks a decline across multiple cognitive domains, including memory, comprehension, language, attention, reasoning, and judgment, profoundly impacting the affected individuals [2]. The epidemiological terrain of AD presents a stark portrayal of its societal impact, with an estimated 6.7 million affected individuals aged 65 and above in the United States alone. Projections paint a worrisome picture, hinting at a potential surge in cases to 13.8 million by 2060 unless significant medical breakthroughs materialize [3]. With its mortality rates and formidable trajectory, AD stands prominently among the leading causes of death, underscoring the pressing need for innovative strategies to alleviate its toll on individuals, families, and healthcare systems at large.

The pathophysiological intricacies of AD involve the accumulation of amyloid-beta (Aβ) plaques and hyperphosphorylated tau protein, instigating neuronal degeneration and neuroinflammation [4-6]. This intricate interplay among Aβ deposition, tau pathology, and neuroinflammation propels disease progression, culminating in synaptic dysfunction, neuronal demise, and cognitive decline [7]. An intricate understanding of these pathways is paramount for devising therapies to decelerate disease advancement. Treatment hurdles in AD persist despite advancements in understanding its cellular and molecular mechanisms [8]. Efforts to develop disease-modifying therapies have faltered due to challenges such as presymptomatic neuronal damage, adverse drug effects, and trial design inadequacies, highlighting the urgent need for focused intervention strategies [9]. The amyloidogenesis pathway, pivotal in AD, offers avenues for targeting toxic amyloidogenic products through diverse interventions. These strategies encompass interventions targeting amyloid protein monomers to impede their conversion into oligomers and the passivation of oligomers and proto-fibrils. Dissociation of toxic forms into monomers or expedited conversion into non-toxic fibrils represents alternative approaches. While the toxicity of fibrils remains contentious, interventions aiming to dissolve fibrils into monomeric entities are deemed desirable [10].

Quantum dots (QDs) emerge as promising nanotechnological tools endowed with unique photo-physical properties. These nano-crystals offer advantages over conventional dyes and imaging modalities, heralding a new frontier in biomedical research. Exploiting the potential of QDs holds immense promise in revolutionizing disease diagnosis and treatment paradigms [11,12]. Their tunable optical properties, biocompatibility, and versatile surface chemistry render them ideal candidates for myriad applications, including targeted drug delivery, bioimaging, and disease biomarker detection [13]. In the therapeutic vacuum of AD, QDs emerge as versatile contenders poised to address the intricate pathophysiological cascades underpinning the disease. QDs intervene at multiple points along the fibril-forming trajectory by preventing the conversion of both monomeric and oligomeric amyloiogenic intermediates into mature fibrils [10]. By delivering therapeutic payloads precisely to diseased tissues while mitigating off-target effects, QDs present a promising avenue for traversing the blood-brain barrier (BBB) and selectively targeting pathological hallmarks of AD, such as Aβ plaques and tau aggregates [12]. This systematic review endeavors to synthesize existing literature to elucidate the theranostic potential of QDs in AD. Through comprehensive analyses of preclinical and clinical studies, we aim to discern the strengths, limitations, and future trajectories of QD-based theranostic approaches, paving the way for translational research and clinical implementation.

## Review

Materials and methods

This systematic review adheres to the Preferred Reporting Items for Systematic Reviews and Meta-Analyses (PRISMA) guidelines, ensuring a rigorous and comprehensive evaluation of studies investigating the theranostic potential of QDs in AD.

Search Strategy

A systematic search strategy was executed across prominent electronic databases, including PubMed, Embase, Hinari, and the Cochrane Library. The search strategy employed a combination of Medical Subject Headings (MeSH) terms and keywords related to QDs and AD. Boolean operators (AND, OR) were utilized to refine the search and identify studies meeting predefined inclusion criteria.

Eligibility Criteria

To ensure the inclusion of studies of utmost quality and relevance, rigorous eligibility criteria were meticulously defined. Studies selected for consideration were required to investigate the theranostic potential of QDs in AD. Additionally, studies must be published in peer-reviewed journals, ensuring academic scrutiny and validation. The temporal scope of the review ranged from inception to January 2024, ensuring the inclusion of the most recent research. Studies lacking adequate data on QDs in AD were deliberately excluded. Furthermore, studies conducted solely on animal models were excluded to prioritize human-relevant findings. Finally, studies published in languages other than English or those lacking full-text availability were excluded to ensure accessibility and comprehensibility for the systematic review process.

Data Extraction and Synthesis

Two independent reviewers conducted an initial screening of titles and abstracts, followed by a detailed assessment of full texts to ensure adherence to inclusion criteria. Any discrepancies between reviewers were resolved through discussion or consultation with a third reviewer. Relevant data, including study design, intervention details, and outcomes, were systematically extracted using a predefined data extraction form.

Data Analysis

A narrative synthesis approach was employed due to anticipated heterogeneity in study designs and outcome measures. Key themes and patterns related to the theranostic potential of QDs in AD were identified and presented. This method ensures a comprehensive and transparent evaluation of the existing literature.

This rigorous methodology forms the foundation for systematically reviewing and synthesizing evidence regarding the theranostic potential of QDs in AD.

Results

Study Selection Process

**Figure 1:**
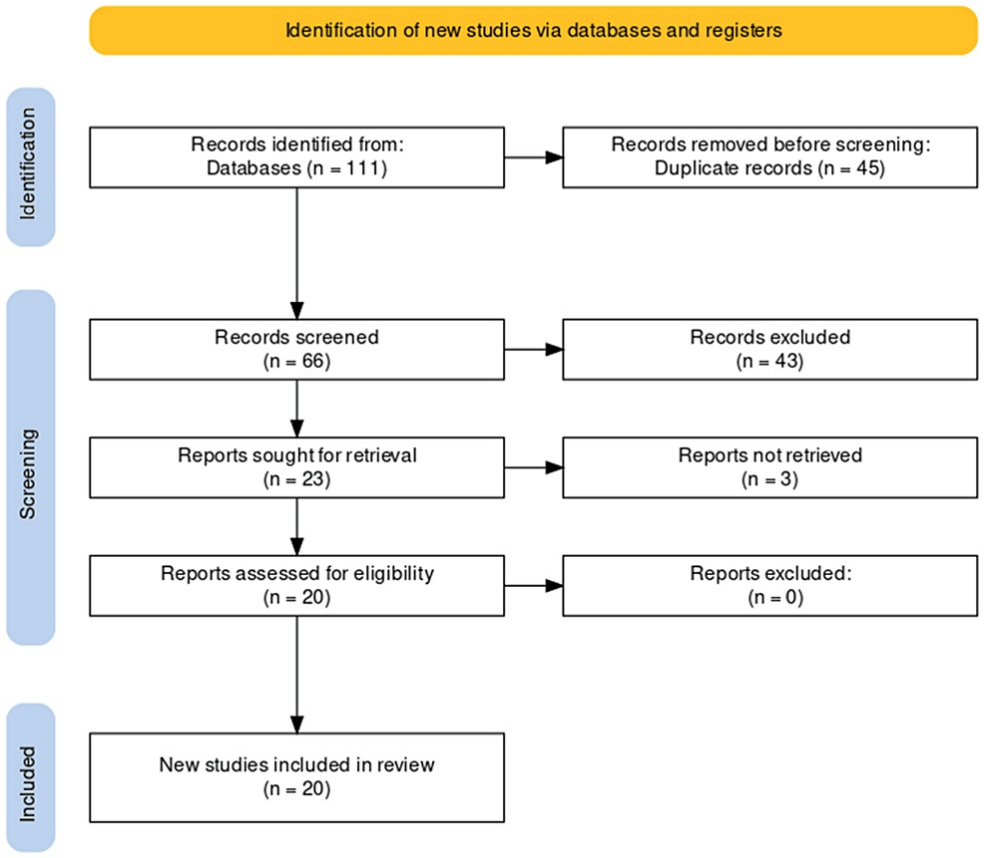
PRISMA diagram showing the study selection process.

Following the PRISMA guidelines, the study selection process was meticulously conducted to ensure transparency and systematicity. Initially, a comprehensive search yielded a total of 111 studies. After removing 45 duplicates, a refined pool of 66 unique studies remained. Screening of titles and abstracts led to the exclusion of 43 records that did not meet predefined relevance criteria. Subsequent evaluation of the full texts of the remaining 23 articles resulted in the exclusion of three reports that did not align with stringent inclusion criteria. Following this rigorous selection process, 20 studies were identified as suitable for inclusion in the systematic review. The study selection process is illustrated in the PRISMA flowchart (Figure 1).

Study Characteristics

The included studies originate from various countries, with the majority coming from the USA and China (seven studies), followed by Japan (three studies), Poland (two studies), and Canada (one study). All the studies are experimental in nature, with the majority focusing on the therapeutic aspect of QDs (13 studies), some on the diagnostic aspect (four studies), and a few exploring the combined theranostic potential (three studies). The table provides information on the type of QDs used, their size, and surface modifications employed in these studies. Several types of QDs have been explored, such as graphene QD (GQDs), carbon QDs (CQDs), cadmium telluride (CdTe) QDs, and molybdenum disulfide QDs, among others. The QD sizes vary significantly, ranging from ultrasmall particles of around 2 nm to larger ones of approximately 30 nm (Table 1).

**Table 1:** Study characteristics of included studies. QD: Quantum Dot, CQD: Carbon Quantum Dot, GQD: Graphene Quantum Dot, CdSe/ZnS QD: Cadmium Selenide/Zinc Sulfide Quantum Dot, CdTe QD: Cadmium Telluride Quantum Dot, Aβ: Amyloid-beta, mBtBDNF: Monobiotinylated Brain-Derived Neurotrophic Factor, MoS2: Molybdenum Disulfide, CND: Carbon Nitride Dot, B-CD: Black Carbon Dot, MPA: Mercaptopropionic Acid

Author	Year	Country	Study type	Theranostic aspect	QD Type	QD size (nm)	Surface modification
Tokuraku et al. [14]	2009	USA	Experimental study	Diagnostic	QDAβ	Not Specified	Aβ
Thakur et al. [15]	2011	USA	Experimental study	Therapeutic	CdSe/ZnS QD	2.5 ± 1.3 nm	Dihydrolipoic acid
Ishigaki et al. [16]	2013	Japan	Experimental study	Therapeutic	QD655	Not Specified	Polyethylene glycol
Głowacz et al. [17]	2015	Poland	Experimental study	Diagnostic	Thiomalic acid-capped QD	Not Specified	Thiomalic acid
Zhao et al. [18]	2015	USA	Experimental study	Diagnostic	QD655	Not Specified	mBtBDNF
Liu et al. [19]	2019	China	Experimental study	Therapeutic	GQD	4.0 ± 0.7 nm	Not Specified
Lin et al. [20]	2020	Japan	Experimental study	Theranostic	Commercially available QDs, type not specified	Not Specified	Not Specified
Koppel et al. [21]	2020	China	Experimental study	Therapeutic	CQD	2.8 nm	Not Specified
Sikorska et al. [22]	2020	Poland	Experimental study	Therapeutic	CdTe QD	3.8 nm	CdTe
Li et al. [23]	2021	China	Experimental study	Therapeutic	MoS2 QD	2.2 ± 0.7 nm	MoS2
Damian Guerrero et al. [24]	2021	USA	Experimental study	Therapeutic	CQD	2-4.2 nm (average 2.7 nm)	Na-Citrate
Tang et al. [25]	2021	China	Experimental study	Therapeutic	GQD	11.4 ± 3.5 nm	Hydroxylation
Zhou et al. [26]	2021	China	Experimental study	Therapeutic	CQD	25 nm	Selenium
Zhang et al. [27]	2022	USA	Experimental study	Therapeutic	CND and B-CD	1.75nm average	Memantine hydrochloride
Lu et al. [28]	2022	China	Experimental study	Diagnostic	CdSe/CdS/ZnS QD	Not Specified	Mercaptopropionic acid (MPA)
Sasaki et al. [29]	2022	Japan	Experimental study	Therapeutic	QD655 and QD605	Not Specified	Not Specified
Minh Quang et al. [30]	2023	USA	Experimental study	Therapeutic	Thiosemicarbazone QD	Not Specified	Not Specified
Walton-Raaby et al. [31]	2023	Canada	Experimental study	Theranostic	GQD7 and GQD28	Not Specified	Not Specified
Bhaloo et al. [32]	2023	USA	Experimental study	Theranostic	GQD	Ag-GQDs: 27.24 ± 5.33 nm, Al-GQDs: 2.79 ± 0.53 nm, rest not specified	Heteroatom doping (Ag, Al, Ce, Fe, Ho, N, Mo, Nd and Tm)
Liu et al. [33]	2023	China	Experimental study	Therapeutic	CQD	2.2 nm	Nitrogen

A summary of the main findings of the included studies is given in Table 2.

**Table 2:** A summary of the main findings of included studies. QD: Quantum Dot, CQD: Carbon Quantum Dot, Aβ: Amyloid-Beta, DHLA: Dihydrolipoic Acid, BDNF: Brain-Derived Neurotrophic Factor, GQD: Graphene Quantum Dot, MSHTS: Microfluidic Diffusional Semiconductor Nanocrystal Tracking System, IAPP: Islet Amyloid Polypeptide, LPS: Lipopolysaccharide, AgNP: Silver Nanoparticle, Msr1: Macrophage Scavenger Receptor 1, AGER: Advanced Glycosylation End Product-Specific Receptor, Aβ-o: Amyloid-Beta Oligomer, FTIR: Fluoroscopic Transform Infrared Spectroscopy, TEM: Transmission Electron Microscopy, HEWL: Hen Egg White Lysozyme, Aβ-m: Amyloid-Beta Monomer, Aβ-f: Amyloid-Beta Fibril, ROS: Reactive Oxygen Species, SeDQD: Selenium-Doped QD, SeCQD: Selenium-Doped CQD, CD: Carbon Dot, B-CD: Black- Carbon Dot, MH: Memantine Hydrochloride, FRET: Förster Resonance Energy Transfer, AChE: Acetylcholinesterase, SF: Straight Filaments, PHF: Paired Helical Filaments, EGCG: Epigallocatechin-3-Gallate, Al-GQD: Aluminum-Doped GQD

Author	Clinical outcome	AD biomarker	Main findings
Tokuraku et al. [14]	Aβ oligomerization and fibrillization using QDs	Aβ42 and Aβ40	QDAβ is a novel nanoprobe for studying Aβ oligomerization and fibrillization in multiple modalities and may be applicable to high-throughput drug screening systems.
Thakur et al. [15]	Effects of DHLA capped CdSe/ZnS QDs on the fibrillation process of Aβ1–42	Aβ1–42	There is a significant change in the morphology of fibrils when Aβ1–42 is mixed or conjugated to DHLA-capped CdSe/ZnS QDs.
Ishigaki et al. [16]	Novel microliter-scale high-throughput screening system for Aβ aggregation inhibitors	Aβ	This novel microliter-scale high-throughput screening system could be applied to the actual screening of Aβ aggregation inhibitors.
Głowacz et al. [17]	Determination of close structural analogs-short-length Aβ peptides	Aβ1-16, Aβ4-16, Aβ4-9, Aβ5-16, Aβ5-12, Aβ5-9, Aβ12-16	Short-length Aβ peptides can be detected using thiomalic acid-capped QDs even at nanomolar concentrations.
Zhao et al. [18]	Trace axonal transport of BDNF	BDNF	QD-BDNF moved essentially exclusively retrogradely, with very few pauses, at a moving velocity of around 1.06 μm/sec.
Liu et al. [19]	Regulatory effects and mechanism of GQDs on Aβ1-42 aggregation.	Aβ1-42	GQDs regulate Aβ1–42 aggregation at a 1:1 mass ratio, inhibiting fibril formation and maintaining non-toxic aggregates. GQDs form a hybrid network with Aβ1–42 via strong interactions instead of keeping it freely soluble.
Lin et al. [20]	Real-time 3D-imaging and inhibition analysis of Aβ, tau, and α-synuclein aggregation utilizing the affinity between QDs and amyloid aggregates.	Aβ, tau and α-synuclein	The study successfully visualized real-time aggregation of Aβ, tau, and α-synuclein using QD nanoprobes coupled with the MSHTS system, revealing differences between amyloid peptides and quantifying inhibition activities of rosmarinic acid.
Koppel et al. [21]	Catalytic effects of LPS on IAPP and Aβ amyloidoses, and further demonstrated their mitigation with zero-dimensional CQDs	Aβ	The study links bacterial endotoxin LPS to amyloid diseases like T2D and AD by speeding up peptide fibrillization. CQDs counteract this by inhibiting Aβ and IAPP, indicating potential as anti-amyloidosis nanomedicine.
Sikorska et al. [22]	Effect of AgNP and CdTeQD on the expression of the Msr1, AGER and Cd36 receptors and Aβ uptake by BV-2 microglial cells	Aβ	AgNP uptake competes with Aβ uptake by microglial cells, impairing aggregate removal. CdTeQD treatment causes the accumulation of proinflammatory Cd36 protein on the cell surface.
Li et al. [23]	Recovery of membrane fluidity by ultrasmall MoS2 QDs	Aβ-o	The study found ultrasmall MoS2 QDs inhibit β-sheet formation in Aβ peptides, maintaining a monomeric state. Confirmed via FTIR spectroscopy and TEM imaging. Computational simulations show reduced Aβ-o size and aggregation inhibition. Suggests therapeutic potential for ultrasmall MoS2 QDs in AD.
Damian Guerrero et al. [24]	Impact of Na–citrate derived CQD introduction on the amyloid-fibril forming tendency of HEWL	HEWL	Na-citrate-derived CQDs halted fibril formation by blocking the conversion of HEWL intermediates and disassembling existing fibrils, transitioning oligomers to monomers.
Tang et al. [25]	Effects of GQDs on the obstruction of the membrane axis of Aβ in Aβ-m, Aβ-o, and Aβ-f	Aβ-m, Aβ-o, and Aβ-f	This study positively implicated GQDs as an effective agent in breaking down the membrane axis of Aβ, thereby circumventing adverse downstream events such as restoring membrane fluidity and offering a potential therapeutic solution for AD.
Zhou et al. [26]	Possible effects of SeCQDs on Aβ aggregation and ROS	Aβ aggregation and ROS	SeDQDs and SeCQDs inhibit Aβ aggregation, a hallmark of AD. SeDQDs have antioxidant properties, while SeCQDs improve memory deficits and reduce Aβ accumulation, inhibiting neuron degeneration.
Zhang et al. [27]	CDs in targeting tau aggregation	Tau aggregation	The kinetics and magnitude of tau aggregation were assessed with CDs. Both B-CDs-MH and B-CDs are potent inhibitors of tau aggregation, with IC50 values of 1.5 ± 0.3 and 1.6 ± 1.5 μg/mL, respectively. These findings have therapeutic significance for delivering MH to target AD pathology in the brain for enhanced efficacy.
Lu et al. [28]	Develop a high-quality CdSe/CdS/ZnS QD-based FRET Aptasensor for the simultaneous detection of Aβ-o and tau protein	Aβ-o and tau protein	A FRET aptasensor employing dual-color CdSe/CdS/ZnS QDs detects AD biomarkers AβO and tau protein simultaneously.
Sasaki et al. [29]	Automated real-time MSHTS for amyloid aggregation inhibitors	Aβ42	Automated MSHTS system is a novel and robust tool that can be adapted to a wide range of compounds and aggregation-prone polypeptides
Minh Quang et al. [30]	Treatment of AD using thiosemicarbazone QDs	Acetylcholinesterase	Four new compounds (N2, N3, N4) designed using QSARKPLS, QSARANN, and QSARSVR models show pIC50 activities comparable to Lead Li 39. They could be potential AChE inhibitors in AD treatment.
Walton-Raaby et al. [31]	Binding sites for GDQ7 and GDQ28 on two forms of Tau protein, SF and PHFs.	SF and PHFs	GQD28 targets unique AD binding sites near protofibril interfaces, including disaggregation sites with EGCG. GQD7 binds broadly, predominantly interacting with PHF6. Potential for detecting, preventing, and disassembling tau aggregates in AD.
Bhaloo et al. [32]	Role of Doped GQDs as Radical Scavengers	ROS scavenging effect	Ten doped GQDs were synthesized for antioxidant properties compared to ascorbic acid. All GQDs showed high biocompatibility (>80% cell viability) and antioxidant abilities in assays. Al-GQDs had the highest reducing power, others showed moderate scavenging success. All GQDs exhibited visible spectrum fluorescence and cell internalization, suggesting clinical applications in AD.
Liu et al. [33]	Effects of nitrogen-doped CQDs on the aggregation process of Aβ1–42	Aβ1–42	Nitrogen-doped CQDs exhibit the capacity to inhibit the Aβ1–42 self-assembly.

Discussion

The profound impact of AD on individuals, families, and healthcare systems underscores the pressing need for innovative therapeutic and diagnostic approaches. QDs emerge as versatile nanotechnological tools that hold immense promise in addressing the multifaceted pathophysiology of AD. Interestingly, the studies encompassed a diverse array of QD types, including GQDs, CQDs, CdQDs, and MoS2QDs, among others. This diversity highlights the versatility of QDs and the potential for tailoring their properties to specific therapeutic targets or diagnostic applications. Moreover, the varying sizes and surface modifications employed in these studies underscore the importance of optimizing QD characteristics to enhance biocompatibility, target specificity, and therapeutic efficacy.

A central theme observed across multiple studies in this review is the ability of QDs to modulate the amyloidogenesis pathway, a pivotal contributor to AD pathogenesis. Several studies have demonstrated the efficacy of QDs in inhibiting the conversion of Aβ monomers and oligomers into mature fibrils, a critical step in plaque formation [19-21,23,25,33]. By maintaining Aβ in a non-toxic, monomeric state or stabilizing intermediates, QDs mitigate the downstream neurotoxic effects associated with fibril accumulation. This intervention at the molecular level holds therapeutic significance, as the disruption of amyloid aggregation could potentially halt or reverse the cognitive decline observed in AD patients [34]. Notably, Liu et al. demonstrated that GQDs effectively regulated Aβ1-42 aggregation at a 1:1 mass ratio, inhibiting fibril formation and maintaining non-toxic aggregates [19]. This finding aligns with the proposed mechanism of QDs intervening along the fibril-forming trajectory to prevent the conversion of amyloidogenic intermediates into mature fibrils. Li et al. demonstrated that ultrasmall MoS2 QDs inhibited β-sheet formation in Aβ peptides, maintaining their monomeric state [23]. Computational simulations corroborated these findings, showcasing reduced Aβ oligomer size and aggregation inhibition, underscoring the therapeutic potential of ultrasmall MoS2 QDs in AD. Similarly, Koppel et al. highlighted the ability of CQDs to mitigate the catalytic effects of bacterial endotoxin LPS on Aβ and IAPP amyloidoses, positioning CQDs as potential anti-amyloidosis nanomedicines [21].

Beyond the amyloidogenesis pathway, Zhang et al. demonstrated the potent inhibitory effects of CNDs and B-CDs on tau aggregation, with promising implications for delivering memantine to target AD pathology in the brain [27]. Similarly, Walton-Raaby et al. investigated the binding sites of GQDs on two forms of tau protein, revealing their potential for detecting, preventing, and disassembling tau aggregates in AD [31]. Their findings revealed that GQD28 targets a unique AD binding site near protofibril interfaces, including a disaggregation site shared with epigallocatechin-3-gallate (EGCG), while GQD7 predominantly interacts with PHF6. These insights hold promise for detecting, preventing, and disassembling tau aggregates in AD. Furthermore, the antioxidant properties of QDs have been investigated in the context of AD pathogenesis. Zhou et al. demonstrated the ability of SeDQDs to scavenge reactive oxygen species (ROS), while SeCQDs exhibited the potential to improve memory deficits and reduce Aβ accumulation [26]. Similarly, Bhaloo et al. synthesized GQDs by heteroatom doping and evaluated their radical scavenging effects, demonstrating high biocompatibility (>80% cell viability) and antioxidant abilities in various assays [32]. Notably, Al-GQDs exhibited the highest reducing power, while others showed moderate scavenging success.

The diagnostic applications of QDs in AD have also garnered significant attention. Studies such as those by Lu et al. and Tokuraku et al. have explored the development of QD-based fluorescence resonance energy transfer (FRET) aptasensors and nanoprobes, respectively, for the simultaneous detection of AD biomarkers such as Aβ oligomers and tau protein [14,28]. Lin et al. successfully visualized real-time aggregation of Aβ, tau, and α-synuclein using QD nanoprobes coupled with the MSHTS system, enabling quantification of inhibition activities of rosmarinic acid [20]. These innovative diagnostic tools hold promise for early and accurate disease detection, a crucial factor in implementing timely interventions and improving patient outcomes.

While the findings presented in this systematic review are promising, it is crucial to acknowledge the challenges and limitations associated with QD-based theranostic approaches in AD. Biocompatibility concerns, particularly for heavy metal-based QDs, necessitate careful consideration and optimization of surface modifications to mitigate potential toxicity. Sikorska et al. reported that CdTe QDs caused the accumulation of proinflammatory CD36 protein on the surface of microglial cells, highlighting the need for rigorous safety assessments [22]. Additionally, the ability of QDs to traverse the BBB remains a critical barrier to their effective delivery to the brain, requiring further investigation into BBB-permeable formulations or alternative administration routes. Moreover, the translation of preclinical findings to clinical settings poses significant hurdles. The complexities of human physiology and the multifactorial nature of AD pathogenesis may introduce unanticipated challenges in translating these findings to clinical practice. Rigorous clinical trials are essential to evaluate the safety, efficacy, and real-world applicability of QD-based theranostic approaches in AD patients.

Despite these challenges, the collective evidence presented in this systematic review underscores the immense potential of QDs as theranostic agents in AD. Their ability to target multiple pathological hallmarks, deliver therapeutic payloads, and facilitate real-time imaging and high-throughput screening positions QDs as versatile tools in the fight against this debilitating disease. Ongoing research and interdisciplinary collaborations between nanotechnologists, neurologists, and pharmaceutical scientists are crucial to overcoming the existing barriers and propelling the clinical translation of QD-based theranostic approaches.

## Conclusions

This systematic review has comprehensively synthesized the existing literature on the theranostic potential of QDs in AD. The findings underscore the versatility of QDs in modulating amyloidogenesis pathways, inhibiting tau protein aggregation, enabling simultaneous detection of AD biomarkers, and exhibiting antioxidant properties. While promising, challenges such as biocompatibility, BBB penetration, and clinical translation warrant further investigation. Nonetheless, the collective evidence highlights the immense potential of QDs as multifaceted theranostic agents capable of addressing the complex pathophysiology of AD. Ongoing research and interdisciplinary collaborations are crucial to overcoming existing barriers and propelling the clinical translation of QD-based theranostic approaches, ultimately paving the way for innovative diagnostic and therapeutic strategies in the fight against this debilitating neurodegenerative disease.
